# Factors Influencing Consultant Knee Surgeons’ Decision Making in Anterior Cruciate Ligament (ACL) Injury Management in Athletes: An International Delphi Study

**DOI:** 10.1007/s40279-026-02480-x

**Published:** 2026-06-25

**Authors:** Greg Young, Nick Dobbin, Neil Jain, Cari Thorpe

**Affiliations:** 1 Department of Health Professions, Faculty of Health and Education, Manchester Metropolitan University, Manchester, M15 6GX UK; 2 Institute of Sport, Manchester Metropolitan University, Manchester, M15 6GX UK; 3 Department of Orthopaedics, The Wilmslow Hospital, 52 Alderley Road, Wilmslow, SK9 1NY UK

## Abstract

**Background:**

Prolonged surgical waiting lists and changing healthcare systems have raised questions about the best approach to anterior cruciate ligament (ACL) injury management in athletes, highlighting the need for consultant knee surgeon consensus.

**Objective:**

The aim was to establish consultant knee surgeon consensus on ACL injury management for athletes.

**Methods:**

Twenty-two male consultant knee surgeons from the United Kingdom (UK) (*n* = 18), Australia (*n* = 2), and Canada (*n* = 2) completed all three rounds of a Delphi survey between April and September 2024, responding to statements developed from the existing literature and clinical expertise.

**Results:**

Median responses ranged from strong agreement to strong disagreement, indicating diverse views, with narrower interquartile ranges in later rounds suggesting increasing consensus. Agreement was observed for rehabilitation‑focused approaches, patient involvement in shared decision making, and clinical factors such as knee stability and concomitant injury, while patient age and level of sport were less consistently prioritised. Disagreement persisted regarding surgical risk, including whether surgery would be required, and graft preferences (autologous versus synthetic). Some items reached full agreement (100%) or showed clustered responses (interquartile range = 0). Most items were stable between rounds (Wilcoxon: *p* = 0.16–1.00), although variability was observed for first-choice management, inclusion of lateral extra-articular procedures, and preferred lateral extra-articular procedure type, despite minimal median change (Wilcoxon: *p* = 0.02–0.09). Kendall’s *W* indicated strong agreement in round 2 (*W* = 0.532, *p* < 0.001) and round 3 (*W* = 0.672, *p* < 0.001).

**Conclusions:**

These findings may support clinicians and athletes in navigating complex treatment decisions, particularly where public healthcare delays challenge conventional surgical pathways.

**Supplementary Information:**

The online version contains supplementary material available at https://doi.org/10.1007/s40279-026-02480-x.

## Key Points

**Table:** 

This study provides international insight from consultant knee surgeons into anterior cruciate ligament (ACL) management across public healthcare systems, highlighting the importance of knee stability and concomitant injury in decision making, with patient choice variably influencing management decisions.
The findings reinforce the value of structured rehabilitation and routine pre‑surgical reassessment, demonstrating that meaningful functional improvement can legitimately influence whether operative management is required.
Despite increasing evidence supporting non‑surgical pathways in selected cases, concern remains regarding rehabilitation‑only management in elite athletes, reflecting persistent uncertainty and variability in clinical practice within high‑performance sport.
The study reflects a move away from rigid, time‑based surgical pathways towards more individualised, function‑based decision making, while also identifying ongoing disagreement around graft selection and the use of lateral extra‑articular procedures, highlighting priorities for future evidence‑informed guidance.

## Introduction

Anterior cruciate ligament (ACL) injuries are among the most common knee injuries in sport and affect athletes at all levels [1]. These injuries are associated with substantial time-loss and long-term consequences, including recurrent instability, secondary meniscal injury, and early onset osteoarthritis [2, 3]. Consequently, increasing research has focused on optimising ACL injury management [4]. In the United States (US), between 200,000 and 250,000 ACL reconstruction (ACLR) procedures are performed annually, with estimated costs of 3 to 7 billion US dollars [5, 6]. In Australia, ACLR and rehabilitation are estimated to cost approximately 15,000 AUD per individual, with total costs projected to reach 314 million AUD by 2030 [7]. Despite frequent surgical intervention, outcomes remain variable, with many individuals reporting persistent symptoms and functional limitations [7]. ACL injury is also associated with a four- to six-fold increased risk of post-traumatic osteoarthritis, irrespective of surgical or non-surgical management [7–9], although this association is not universal [10].

As the primary stabiliser of anterior tibial translation, the ACL contributes to rotational control of the knee during dynamic tasks such as changing direction [11]. Injury to the ACL disrupts these functions, alters knee kinematics, and increases the risk of secondary injury [12]. ACLR is commonly pursued in athletes due to perceived restoration of stability, predictable return-to-sport timelines, and assumptions of improved long-term joint protection [8, 13, 14]. This paradigm is increasingly challenged, with rehabilitation suggested as a first-line management strategy [8]. Contemporary evidence suggests that both surgical and non-surgical approaches may be appropriate given comparable return-to-sport rates [15], with decisions requiring consideration of patient characteristics, sport demands, treatment goals, rehabilitation access, symptom progression, and response to pre-operative rehabilitation [8, 17].

High-quality comparative studies have contributed to this shift in management, with randomised controlled trials (RCTs), including KANON [15, 18] and COMPARE [19], demonstrating that structured rehabilitation with the option for delayed reconstruction yields similar functional outcomes to early ACLR at mid-term follow-up [20, 21]. A 2024 systematic review reported comparable rates of post-traumatic osteoarthritis between surgically and non-surgically managed patients [22]. These findings challenge the assumption that early surgical intervention is necessary for all athletes and support a more individualised approach, even at the elite sport level [23]. However, clinical practice does not consistently reflect this evidence. Filbay et al. [24] report that many clinicians continue to present ACLR as the preferred option, often without fully communicating that similar outcomes may be achieved with rehabilitation alone. This highlights the influence of clinician preference, experience, and perceptions of return-to-sport risk.

Return-to-sport is a key consideration in ACL management and often drives treatment decisions. Traditionally, return-to-sport timelines following ACLR have been guided by time-based milestones, typically 6–12 months. [16, 25] However, early return (< 6 months) to sport in elite European footballers has shown longer playing careers, albeit with higher reinjury rates [26]. Criterion-based approaches incorporating strength, functional performance, and neuromuscular control have therefore been proposed as indicators of return-to-sport readiness [14, 27]. Athletes meeting objective return-to-sport criteria demonstrate substantially lower reinjury risk, including an 84% reduction in subsequent ACL injury [14], and have greater odds of returning to pre-injury playing levels [27]. However, implementation remains inconsistent, and the evidence base is limited by small samples, single sex cohorts [27], and limited synthesis [28]. Many athletes return without meeting recommended criteria, while others who meet criteria fail to return, illustrating a gap between evidence and clinical decision making [28, 29]. Ardern et al. [16] reported that, while 81% of individuals returned to sport, only 55% returned to competitive participation, leaving the value of return-to-sport criteria uncertain.

Secondary injury risk also complicates decision making. Both surgical and non-surgical approaches are associated with subsequent ACL and meniscal injuries affecting both knees [30]. Evidence regarding the protective effect of ACLR is mixed, with some studies reporting reduced risk of secondary meniscal injury, particularly with early ACLR [31, 32], whereas others report comparable or increased rates of ipsilateral injury following surgery [33]. Long-term cohort data suggest substantial secondary pathology following both approaches, with activity levels, rehabilitation quality, and intervention timing influencing outcomes [31, 34]. Sanders et al. [31] reported 37.4% of conservatively managed patients developed secondary meniscal tears compared with 8.2% of surgically managed patients over a mean follow up period of 13.7 years. Despite improved knee stability, nearly 30% of patients undergoing ACLR sustained a subsequent non-contact ACL injury within 2 years, with most affecting the contralateral knee [34]. Beyond secondary injury, longer term joint health outcomes also differ, with Kessler et al. [30] reporting higher rates of osteoarthritis in surgically managed patients compared with those managed conservatively (42% versus 25%). This heterogeneity across secondary injury and degenerative outcomes complicates individualised decision making and the provision of clear, evidence-informed advice. Further uncertainty arises from ongoing debate regarding graft selection, with some evidence favouring hamstring tendon autografts and other studies reporting higher return-to-sport rates with bone-patellar tendon-bone (BPTB) grafts in high-demand athletes [24, 35]. Emerging interventions, including biological augmentation and cross-bracing protocols, have also shown promising early results, further expanding management options available to clinicians [36, 37].

When considering factors influencing ACL injury management, the evidence base presents a complex and sometimes conflicting picture. While both surgical and non-surgical pathways are supported in specific contexts, factors such as joint laxity, instability episodes, and functional outcomes may explain why some individuals “cope” with ACL deficiency [38]. However, there remains limited consensus on how these factors should be prioritised or integrated into decision making, with decisions often influenced by clinician experience, evidence interpretation, and contextual constraints rather than a consistent framework [32, 39]. This uncertainty is particularly relevant in athletes, where return-to-sport goals, reinjury risk, long-term joint health, and patient expectations must be balanced. Such findings are reflected in a recent Delphi study [40], that discussed key factors influencing decision making, including concomitant injury, participation in cutting or pivoting sports, and persistent instability. These experts also recommended a period of rehabilitation regardless of management strategy; however, findings were largely derived from clinicians in private healthcare settings and reflected interpretation of a limited evidence base. Consequently, there remains limited understanding of how these factors are weighted across private and public healthcare settings, particularly given increasing service demand and prolonged waiting times for ACLR. This highlights the need for further work to explore how clinicians integrate these considerations in practice and whether consistency exists across different healthcare environments and athletic populations.

Given the ongoing uncertainty, there is a need to better understand how experienced clinicians integrate multiple factors when making ACL management decisions. A Delphi methodology is well-suited to exploring expert consensus where evidence is heterogeneous. Therefore, this study aimed to determine the level of consensus among consultant knee surgeons regarding factors influencing ACL management decisions in athletes.

## Methods

### Development of Round 1 Statements

Round 1 statements were developed based on clinical experience and a non-systematic search of relevant literature (key findings are summarised in the introduction section). Searches were conducted in PubMed, Medline, CINAHL, and SportDiscus, with reference list screening used to identify additional relevant studies. Key publications known to the authors were also included where aligned with the study aims. This approach is consistent with Delphi methodology, in which initial item generation reflects available evidence and expert judgement rather than comprehensive evidence synthesis. A systematic review was therefore not undertaken, as the intent was to capture a broad range of clinically relevant factors influencing ACL injury management, consistent with the exploratory nature of the study. Item development was further informed by the multidisciplinary expertise of the author team, including a physiotherapist, physiologist, consultant knee surgeon, and specialist trainee doctor in sport and exercise medicine, supporting alignment between published evidence and real-world clinical decision making. A summary of the evidence informing item development is provided in Supplement 1 in the electronic supplementary material.

### The Delphi Process

#### Study Design

This study used a three-stage modified Delphi method, with an online survey distributed to an expert panel via email between April and September 2024. The study was conducted and reported in accordance with Conducting and REporting of DElphi Studies (CREDES) guidelines (Supplement 2; see the electronic supplementary material) [41]. Ethics approval was granted by the Faculty of Health and Education Research Ethics and Governance Committee at Manchester Metropolitan University (No. 63076; 02/09/2024).

#### Expert Panel Selection

A total of 110 participants (male, *n* = 101; female, *n* = 9) from Australia (*n* = 22), Canada (*n* = 24), and the United Kingdom (UK) (*n* = 64) were contacted via email and invited to participate using purposive sampling to capture a broad representation of consultant knee surgeons across the three countries. Based on country-specific databases (e.g. National Ligament Registry), it was estimated that 1100–1800 consultant knee surgeons practise across these regions, with approximately 20% managing more than 40 ACL injuries annually, yielding an estimated sampling frame of 220–360 surgeons. Accordingly, 110 invitations represented approximately 31–50% of the eligible population. These countries were selected due to comparable healthcare systems, clinical training pathways, and scope of practice relevant to ACL management. Recruitment was limited to English-speaking countries due to resource constraints.

Participants who appeared to meet the inclusion criteria and had publicly available contact information (*n* = 110) received the survey link, participant information sheet, and consent form. Eligibility criteria included a minimum of 5 years’ experience as a consultant, a period considered sufficient to demonstrate expertise in ACL surgical management through operative volume, exposure to variation in injury presentation and graft choice, and reflection on medium- to longer-term outcomes appropriate for Delphi consensus. Participants were also required to manage at least 40 ACL injuries per year and have prior experience within a public health system. Individuals who did not meet these criteria, including non-consultant grades and non-surgical clinicians, were excluded.

An a priori power calculation using Kendall’s *W* coefficient [42] estimated that a minimum sample of 15–20 participants was required to achieve a 75% level of agreement, consistent with previous literature [43]. To account for potential attrition and maximise external validity, 110 participants were invited using publicly available information from key publications, NHS trusts, recognised orthopaedic centres, and individuals known to the research team, reflecting response rates between Delphi rounds reported previously. [44]

#### Procedures

Prior to the commencement of the Delphi study, the steering committee (the authors) reviewed the existing evidence and developed the initial survey. During this process, distinctions between athletes and elite athletes were considered, and it was deemed necessary to differentiate these groups. In this study, an athlete was defined as an individual of young or adult age who engages in regular exercise training and participates in official sports competition [45], at a level below that described by Swann et al. [46] as semi-elite, competitive elite, successful-elite, or world-class elite. Under Swann et al.’s [46] framework, semi-elite athletes compete and succeed at regional, university, or semi-professional levels, have less than 2 years of experience at these levels, and participate in sports ranked outside the national top ten with global and televised reach. For the purposes of this survey, all levels above semi-elite were categorised as elite.

The steering committee also convened between rounds to discuss findings. The study was conducted over 6 months, with approximately 2-month intervals between rounds. Following each round, responses were exported from the Joint Information Systems Committee (JISC, Version 3) to Microsoft Excel (Version 16.96.1) for analysis. Between rounds 2 and 3, participants received an anonymised summary of the results.

Round 1 collected data on participants’ qualifications, years of experience, and location of clinical practice. Participants then completed a survey derived from the literature using a five-point Likert scale (1 = strongly agree, 2 = agree, 3 = neither agree nor disagree, 4 = disagree, and 5 = strongly disagree). Open-text boxes were included to capture additional feedback and suggestions for new items, which informed revisions to the round 2 questionnaire for clarity. No further changes were made between rounds 2 and 3, enabling assessment of stability of agreement and consistency across respondents. The round 1 survey and the round 2/3 questionnaires are available in Supplements 3 and 4, respectively (see the electronic supplementary material).

### Statistical Analysis and Reporting

Data were analysed using SPSS (Version 29.0.2.0, IBM, USA) and Microsoft Excel (Version 16.96.1) to assess consensus within and between rounds, including only participants who completed rounds. This approach ensured that changes in agreement reflected within-participant response variation rather than attrition effects. For each Delphi item (except question 12), the median and interquartile range (IQR) were calculated, with an IQR < 1 indicating a strong consensus. Percentage agreement was also calculated for participants selecting agree or strongly agree (Likert scores 1 or 2) and disagree or strongly disagree (scores 4 or 5). General agreement was defined as the percentage of participants selecting the most frequently chosen response. Question 12 was analysed as the proportion of respondents ranking factors influencing ACL management from most important (1) to least important (5). Visualisations were produced using RStudio (Version 2025.05.0 + 496), with the code used provided in Supplement 5 (see the electronic supplementary material).

Agreement between rounds was assessed using the Wilcoxon signed-rank test, comparing paired responses for each item between rounds 2 and 3. Exact test statistics and *p* values were interpreted on a scale of compatibility (1.0) to incompatibility (0.0) with the null model for each statistical test.

Kendall’s coefficient of concordance (*W*) was used to assess agreement among participants within each round across all questions. Thresholds were interpreted as follows: 0.01–0.10 = negligible agreement; 0.11–0.30 = weak agreement; 0.31–0.50 = moderate agreement; 0.51–0.70 = strong agreement; and 0.71–1.00 = very strong agreement [48]. Kendall’s *W* therefore provided a global indicator of the consistency with which participants ranked items within each round.

### Steering Committee Characteristics and Equity, Diversity, and Inclusion Statement

The author group comprised a physiotherapist with over 10 years’ experience managing ACL patients, a consultant knee surgeon with 11 years’ experience, a sport and exercise physiologist with 5 years’ experience assessing ACL patients during rehabilitation, and a specialist trainee doctor undertaking a master’s degree in sport and exercise medicine. Representing both sexes, the authors developed the first round of the study based on clinical expertise and a review of the literature.

### Patient and Public Involvement

Patients and the public were not involved in the design, conduct, reporting, or dissemination of this study.

## Results

Thirty-one male consultant knee surgeons responded in round 1 (UK = 27, Canada = 2, Australia = 2), representing 29% of those contacted and 9–15% of the estimated sampling frame. In round 2, 23 participants completed the survey, of whom 22 (UK = 18, Canada = 2, Australia = 2) also completed round 3, equating to 20% of those contacted and 6–10% of the sampling frame. Analyses were based on the 22 consultant knee surgeons who performed a minimum of 40 ACLRs per year and had at least part-time experience within a public-sector health service. Participants had between 5 and 30 years of experience managing ACL injuries (mean ± standard deviation 17 ± 7 years). Experience distribution was 12.5% (5–10 years), 28.1% (11–15 years), 31.3% (16–20 years), 15.6% (21–25 years), and 12.5% (> 26 years).

**Fig. 1:**
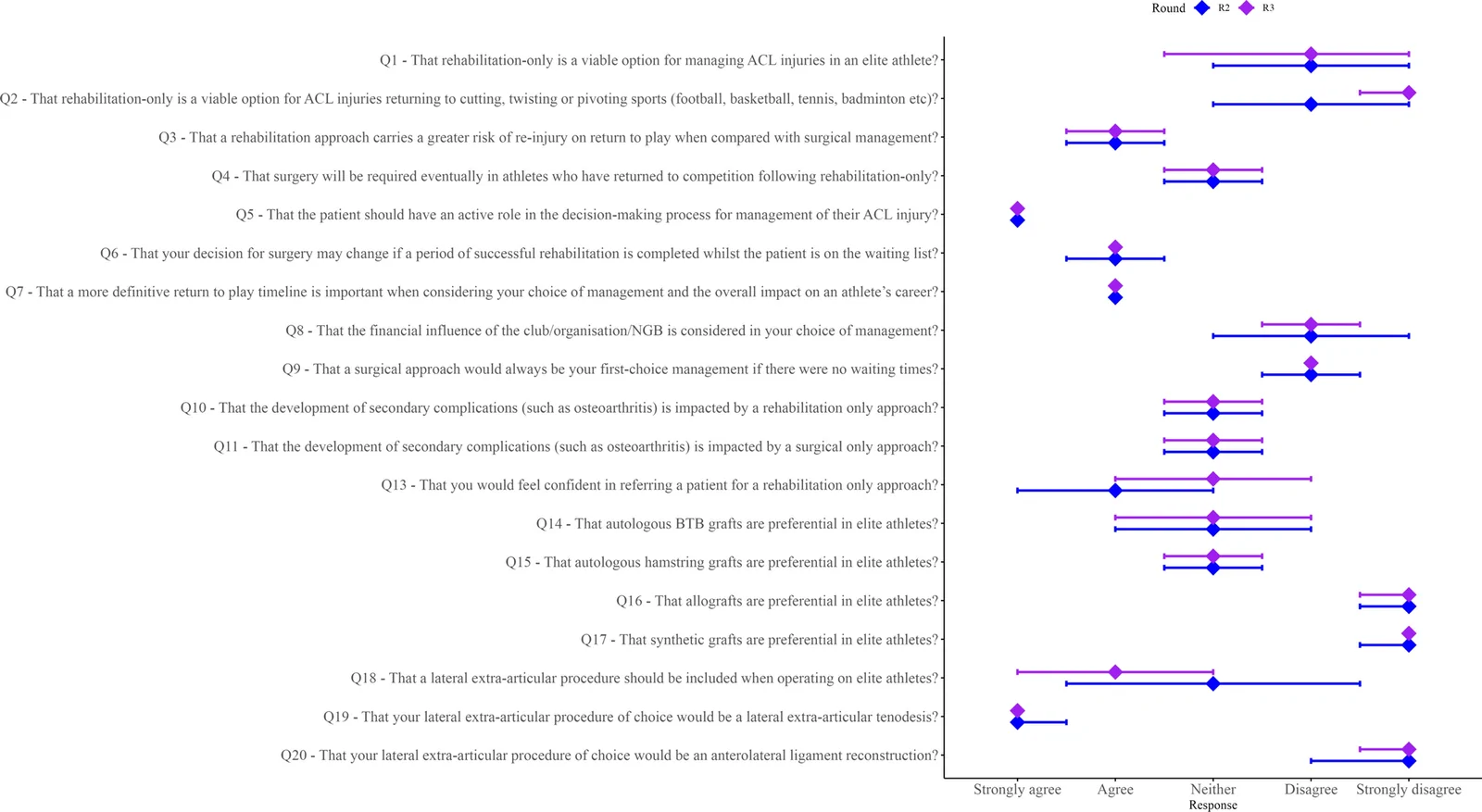
Median and interquartile range for each question per round. ACL, anterior cruciate ligament; Q, question; R, round; BTB, bone-tendon-bone; NGB, national governing body

All Delphi survey questions are presented in Fig. 1 and Table 1 and are referred to as “Q” hereafter. Responses to rounds 2 and 3 are provided in Supplement 6 (in the electronic supplementary material). Median responses across survey items ranged from strong agreement (median = 1) to strong disagreement (median = 5), demonstrating considerable diversity in participant opinion (Fig. 1). Strong agreement was observed for Q5 in both round 2 (blue) and round 3 (purple), with a median score of 1 and a narrow IQR. General agreement (median = 2) was evident for Q3, Q6, and Q7 across both rounds, and for Q13 (round 2) and Q18 (round 3). Neutral responses (median = 3) were observed for several items across both rounds (Q4, Q10, Q11, Q14, Q15), although Q18 in round 2 demonstrated substantial dispersion around this median. Disagreement (median = 4) was observed for Q1, Q8, and Q9 across both rounds, as well as for Q2 in round 2, while strong disagreement (median = 5) was evident for Q16, Q17, and Q20.

**Table 1:** Consensus level for each question

	To what extent do you agree or disagree that…	Strongly agree/agree		Strongly disagree/disagree		Overall agreement	
		R2	R3	R2	R3	R2	R3
1	Rehabilitation-only is a viable option for managing ACL injuries in an elite athlete?	23%	32%	68%	68%	35%	35%
2	Rehabilitation-only is a viable option for ACL injuries returning to cutting, twisting or pivoting sports?	18%	23%	68%	77%	35%	52%
3	Rehabilitation approach carries a greater risk of re-injury on return to play when compared with surgical management?	86%	100%	5%	0%	48%	61%
4	Surgery will be required eventually in athletes who have returned to competition following rehabilitation only?	41%	36%	23%	18%	35%	43%
5	Patients should have an active role in the decision-making process for management of their ACL injury?	100%	100%	0%	0%	83%	91%
6	Your decision for surgery may change if a period of successful rehabilitation is completed whilst the patient is on the waiting list?	73%	86%	5%	9%	48%	74%
7	A more definitive return-to-play timeline is important when considering your choice of management and the overall impact on an athlete’s career?	86%	95%	0%	0%	65%	83%
8	The financial influence of the club/organisation/NGB is considered in your choice of management?	14%	9%	73%	91%	43%	61%
9	A surgical approach would always be your first-choice management if there were no waiting times?	23%	14%	50%	82%	39%	74%
10	The development of secondary complications (such as osteoarthritis) is impacted by a rehabilitation-only approach?	36%	32%	23%	23%	39%	43%
11	The development of secondary complications (such as osteoarthritis) is impacted by a surgical-only approach?	23%	27%	32%	14%	43%	57%
13	You would feel confident in referring a patient for a rehabilitation-only approach?	55%	50%	32%	27%	43%	43%
14	Autologous BTB grafts are preferential in elite athletes?	45%	45%	32%	36%	35%	39%
15	Autologous hamstring grafts are preferential in elite athletes?	14%	9%	36%	45%	48%	43%
16	Allografts are preferential in elite athletes?	5%	5%	91%	95%	61%	70%
17	Synthetic grafts are preferential in elite athletes?	0%	0%	100%	100%	70%	83%
18	Lateral extra-articular procedure should be included when operating on elite athletes?	50%	64%	32%	18%	26%	39%
19	Your lateral extra-articular procedure of choice would be a lateral extra-articular tenodesis?	87%	94%	0%	0%	50%	72%
20	Your lateral extra-articular procedure of choice would be an anterolateral ligament reconstruction?	13%	6%	73%	82%	56%	56%

**Fig. 2:**
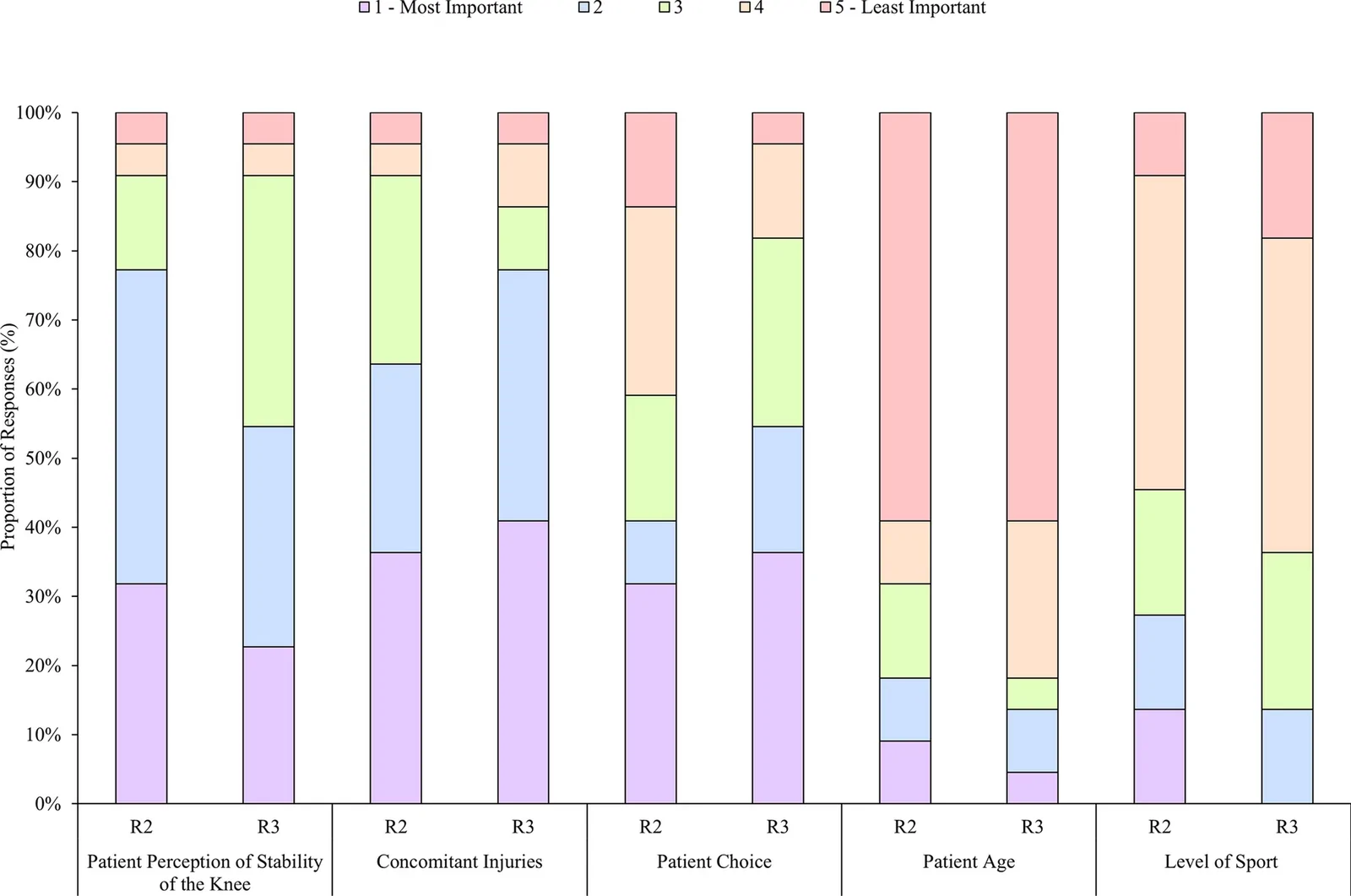
Proportion of respondents who ranked key features that influence the overall management choice. *R* round

Results for question 12 can be found in Fig. 2

ACL, anterior cruciate ligament; R, round; BTB, bone-tendon-bone; NGB, national governing body

IQR values provide insight into the level of consensus, with narrower IQRs indicating tighter clustering of responses and greater agreement among experts (Fig. 1). Consistent with Delphi methodology [19, 21, 23], IQR values below 1 were interpreted as evidence of strong consensus. Several items met this threshold, including Q5 and Q7 in both rounds 2 and 3, which demonstrated an IQR of 0, indicating unanimous or near-unanimous agreement. IQR values for Q3, Q4, Q10, Q11, and Q15–Q17, as well as Q19 and Q20, were at or below 1 across both rounds, reflecting robust clustering around the median. The widest IQRs were observed for Q1, Q13, Q14, and Q18.

Overall, the majority of items showed similar or decreased IQRs across rounds, suggesting that consensus generally increased as the Delphi process progressed. However, a small number of items retained higher IQR values, indicating ongoing divergence in expert opinion on these topics.

For Q12, perceptions of knee stability, concomitant injury, and patient choice were ranked as the most important factors in both rounds. Patient age and level of sport were consistently ranked as less important. Most factors demonstrated substantial variability, with some experts ranking them as least important while others ranked the same factors as most important, with the exception of level of sport in round 3 (Fig. 2).

**Fig. 3:**
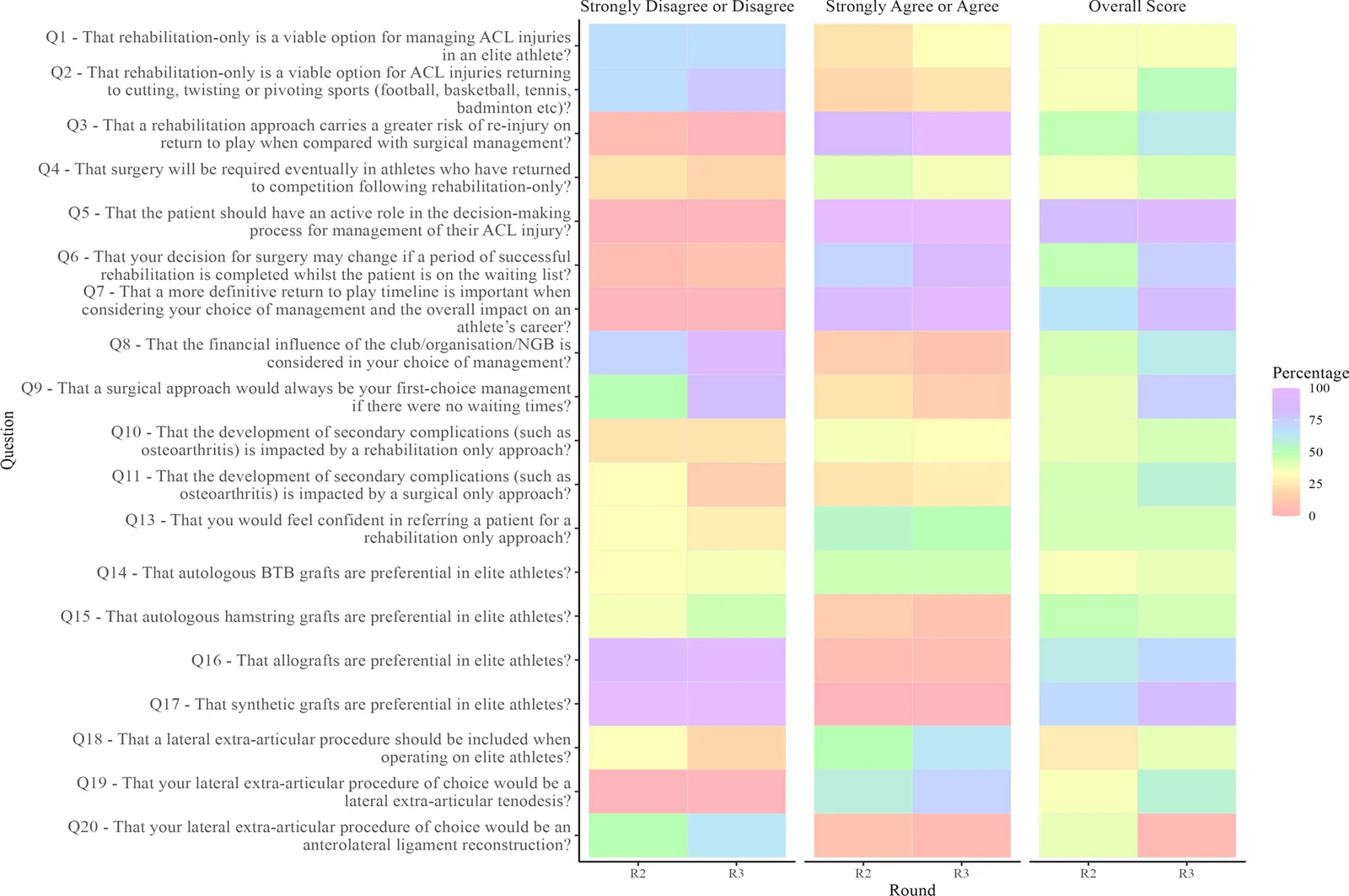
Agreement levels by question and round with the colour reflecting the extent of agreement. ACL, anterior cruciate ligament; Q, question; R, round; BTB, bone-tendon-bone; NGB, national governing body

Percentage-based consensus metrics provided further insight into expert agreement and divergence (Table 1, Fig. 3). Agreement, defined as the proportion selecting “agree” or “strongly agree”, was unanimous for Q5 in both rounds (100%), reflecting strong alignment. Agreement was also observed for Q3 in round 3 (100%) and Q7 in rounds 2 and 3 (86% and 95%). Agreement was identified for Q6 in round 3 (86%), Q13 in round 2 (55%), Q19 in rounds 2 and 3 (87% and 94%), and Q18 in round 3 (64%).

In contrast, several items showed low agreement. Q9 demonstrated agreement levels of 23% and 14%, alongside disagreement levels of 50% and 82% in Rounds II and III. Q10 showed agreement levels of 36% and 32%. Disagreement levels were 91% and 95% for Q16 and 100% in both rounds for Q17. Q14 showed disagreement levels of 32% and 36% and an agreement level of 45% in both rounds, indicating a mixed response pattern. Overall, these findings highlight areas of both robust consensus and ongoing debate in ACL injury management, as summarised in Fig. 3.

Overall agreement, irrespective of direction, ranged from 26 to 91%. The highest convergence was observed for Q5 (83% to 91%), Q7 in round 3 (83%), and Q17 in round 3 (83%), whereas Q18 in round 2 (26%), Q1 in rounds 2 and 3 (35%), and Q14 in round 2 (35%) reflected marked divergence. Several items demonstrated increased overall agreement across rounds (strongly agree/agree or strongly disagree/disagree), including Q3 (48% to 61%), Q4 (35% to 43%), and Q6 (48% to 74%). As analyses were restricted to participants who completed both rounds (*n* = 22), these changes reflect within-participant shifts in responses consistent with iterative feedback and refinement throughout the Delphi process. This approach captures both convergent and polarised expert opinion over time.

Wilcoxon signed-rank tests were performed for each question. Most items demonstrated compatibility with the null hypothesis, indicating stable views across rounds (Q1–Q8, Q10, Q11, Q13–Q17, Q20; *p* = 0.16–1.00). A small number of items showed greater incompatibility with the null hypothesis (Q9, Q18, and Q19; *p* = 0.09, 0.02, and 0.08, respectively), suggesting potential, though inconclusive, shifts in opinion. For Q12, compatibility between rounds was observed for patient perceptions (*p* = 0.135), concomitant injury (*p* = 0.525), and patient age (*p* = 0.293). Greater incompatibility was evident for patient choice (*p* = 0.085) and level of sport (*p* = 0.061).

Kendall’s coefficient of concordance (*W*) was used to assess overall agreement among participants across all items within each round. Results showed moderate agreement in round 2 (*W* = 0.532, *χ*^2^ (18) = 143.596, *p* < 0.001) and stronger agreement in round 3 (*W* = 0.672, *χ*^2^ (18) = 205.751, *p* < 0.001). The increasing values from round 2 to round 3 suggest a successful convergence of expert opinion as the Delphi process progressed.

## Discussion

This study aimed to determine whether consensus exists among consultant knee surgeons regarding ACL injury management. Existing literature reports conflicting findings on return-to-sport and reinjury rates, with limited agreement on the factors influencing management decisions.

The surgeons in this study agreed that successful rehabilitation while awaiting surgery could influence the decision to proceed, supporting reassessment by a surgeon or physiotherapist prior to operative intervention. This aligns with rehabilitation‑first guidelines [8, 10, 17] and findings by Shen et al. [48], who reported that 50% of patients undergoing rehabilitation ultimately avoided surgery. Similarly, the KANON [5, 18] and COMPARE [19] trials demonstrated that 50–61% of individuals engaging in structured rehabilitation were able to avoid ACLR. Pre‑surgical reassessment may therefore reduce unnecessary procedures, associated risks, and healthcare costs, particularly given evidence suggesting that ACLR is not always cost‑effective [20, 21]. Implementation may be constrained by time and resource pressures in busy clinical environments, as well as limited access to experienced physiotherapists through appropriate referral pathways. Interestingly, the relative importance assigned to key decision‑making factors varied across rounds. Level of sport and patient age were not consistently ranked as the most influential factors, despite their frequent emphasis in the literature. Instead, knee stability and concomitant injuries were prioritised, suggesting that clinical presentation may be weighted more heavily than demographic or contextual factors in management decisions. This pattern may reflect a shift towards functionally driven decision making, although further exploration is warranted.

Despite growing evidence that some individuals can return to elite-level sport following non-surgical management [15, 23], 68% of consultant knee surgeons in this study did not view rehabilitation alone as a viable option for elite athletes. This suggests that surgical management remains the predominant approach by surgeons in an elite sport context, consistent with findings from Filbay et al. [24], who reported that surgery is commonly presented as the preferred treatment option. However, substantial variability in clinical opinion was evident. Approximately one in three surgeons considered rehabilitation a viable option for elite athletes, and almost one in four considered it appropriate for participants in cutting and pivoting sports, highlighting heterogeneity in contemporary decision making and emerging divergence in clinical perspectives [7].

This variability may reflect differences in interpretation of the evolving evidence base, clinical experience, and perceived risk profiles. As reasons underpinning decision making were not directly explored, these explanations warrant further investigation. In elite sport, minimising time lost from competition is a key priority, and surgical management may therefore be perceived as offering a more predictable return-to-play timeline compared with rehabilitation alone [8]. Notably, in the RCT by Frobell et al. [18], which supports an initial conservative management approach, approximately 50% of participants underwent delayed ACLR within 2–5 years. This may pose challenges for elite athletes depending on career stage, contract status, competition cycles, and life stage [8]. Consistent with this, 95% of experts in round 3 identified a more predictable return-to-play timeline following surgery as an important factor in decision making.

In contrast to traditional assumptions, level of competition did not consistently emerge as the dominant determinant of management choice. While elite sporting demands are often assumed to necessitate surgical intervention, the observed variability suggests that surgeons may weigh sport-specific demands alongside other clinical factors rather than viewing them in isolation. Similarly, patient age showed considerable variability in perceived importance, indicating that chronological age alone may be less influential than previously assumed when considered alongside functional status, injury characteristics, and rehabilitation potential.

Although injury-related time loss can have substantial financial implications for sports organisations [49], both directly (e.g., healthcare costs) and indirectly (e.g., league standing and sponsorship) [50], financial considerations did not appear to be a primary driver of decision making in this cohort. Indeed, 91% of experts agreed that organisational financial pressures do not influence the choice between rehabilitation-only approaches and ACLR. It should be noted, however, that this study focused on clinicians managing athletes rather than the general population, where cumulative healthcare costs may be greater. Instead, variation in practice may reflect differing levels of confidence in rehabilitation pathways [51], with only approximately half of experts reporting comfort in referring patients for non-surgical management. This may be related to greater familiarity with surgical outcomes and perceptions of increased predictability following ACLR, particularly in elite sporting environments [16], as well as concerns regarding non-operative management, including perceived risk of secondary injury [32, 39]. As these factors were not directly examined, they cannot be confirmed within the current dataset and warrant further investigation. Notable variability was also observed in the importance assigned to patient preference. Although shared decision-making is widely endorsed within clinical guidelines, the dispersion of responses suggests inconsistency in how strongly patient choice is incorporated into treatment decisions. This may reflect tension between patient autonomy and perceived clinical risk, particularly in high-performance contexts. As the operationalisation of patient preference was not explored in this study, further research is required to understand how clinicians balance these considerations in practice.

Importantly, these findings should be interpreted in the context of an evolving evidence base. Although rehabilitation-first approaches and emerging strategies such as cross-bracing protocols and bridge-enhanced ACL repair are increasingly recognised in the literature [8, 18, 19, 37, 52], with some studies suggesting comparable outcomes between surgical and non-surgical pathways in selected populations, the translation of this evidence into clinical practice is likely to be gradual. As such, the present findings may reflect a snapshot of contemporary clinical opinion that warrants a longitudinal follow-up. Perspectives may also differ between athlete groups, as well as between surgeons and rehabilitation clinicians, and may be influenced by uncertainties regarding long-term outcomes, reinjury risk, and the availability of experienced rehabilitation support.

## Research Implications

This study identifies several key areas requiring further research. First, there remains a lack of consensus regarding secondary complications following ACL injury. Both the present findings and those of Diermeier et al. [40] highlight ongoing uncertainty about the long‑term comparative risks of secondary complications following ACLR versus rehabilitation‑only management, reinforcing the need for high‑quality longitudinal studies that account for patient characteristics, activity level, and rehabilitation exposure. While most consultant knee surgeons in this study did not consider rehabilitation‑only management viable for elite athletes, approximately one in three did, reflecting variation in clinical opinion and alignment with an evolving evidence base. Future research should therefore focus not only on clinical outcomes, but also on identifying which athletes may be suitable for rehabilitation‑only approaches, incorporating sport‑specific demands, functional capacity, and contextual factors. There is also a need to better understand how emerging evidence is translated into clinical practice and potential barriers to adoption.

The findings further demonstrate divergence in decision making that is not consistently aligned with traditional assumptions. Factors such as age and level of competition, commonly cited as key determinants of management strategy, were not uniformly prioritised, suggesting that clinical presentation, including knee stability and concomitant injury, may exert greater influence than demographic or sporting characteristics alone. Further research is required to clarify how these factors interact and to support more individualised treatment decisions. Finally, graft selection and the use of lateral extra‑articular procedures remain areas of non‑consensus in both the literature and among experts, indicating the need for additional long‑term outcome studies. Although allografts and synthetic grafts are not recommended as first‑line options for elite athletes, they may be appropriate in complex cases, specific individual circumstances, or when donor‑site morbidity is a concern [53], while the choice between BPTB and hamstring grafts remains debated and influenced by surgeon experience, prior outcomes, and patient‑specific factors [35].

### Strengths

This study involved highly experienced consultant knee surgeons, with a mean of approximately 18 years’ experience and a minimum annual workload of 40 ACLRs. Inclusion of participants from the UK, Australia, and Canada ensured that the findings were not confined to a single geographic region or healthcare system, thereby enhancing external validity. The questionnaire was developed by a multidisciplinary team comprising a specialist trainee doctor in sport and exercise medicine, a consultant sports knee surgeon, a sport and exercise physiologist, and an experienced musculoskeletal physiotherapist.

A further strength was the three‑round Delphi design, which enabled refinement of questionnaire items and assessment of consensus across multiple stages. The findings align with previous research [38], supporting their robustness. Attrition bias was relatively low (28%) compared with other Delphi studies [44], and over‑recruitment in the first round ensured that a sufficient sample size was retained throughout the study.

### Limitations

The online-only format limited opportunities for real-time discussion between participants, which may have enriched the consensus-building process. Several items received a high proportion of “neither agree nor disagree” responses, suggesting that some questions may have been unclear or too complex to resolve using a Likert-scale format. Of the 110 experts contacted, 31 responded and 22 completed the full Delphi process. This introduces the potential for self-selection bias, as well as a gender bias, and therefore the findings may not fully represent the views of the broader consultant knee surgeon population. Future studies should aim to improve gender representation through targeted recruitment strategies, such as oversampling female surgeons, direct engagement with professional networks and specialist societies, and stratified sampling approaches.

Round 1 statements were informed by a non-systematic review of the literature and clinical expertise, which may have constrained the scope of the initial Delphi items. A broader and more systematic evidence review, alongside patient and public involvement, could enable a more comprehensive exploration of factors influencing ACL management. Involvement of additional stakeholders, including rehabilitation professionals such as physiotherapists, would also provide a more balanced perspective on rehabilitation pathways, secondary complications, return-to-sport timelines, and reinjury risk, potentially reducing bias towards surgical management.

Geographic representation was limited, with 82% (18/22) of participants based in the UK and no representation from Africa, Asia, South America, the US, continental Europe, or the Middle East, limiting external validity. This lack of input from surgeons practicing in low- and middle-income countries and the absence of female voices mean that the findings may not fully reflect the diversity of perspectives within the global orthopaedic community. This limitation is common in consensus research [45], and highlights important priorities for future studies.

## Conclusion

This study found that while many surgeons do not consider rehabilitation‑only management appropriate for elite athletes participating in pivoting sports, a notable proportion did, indicating ongoing disagreement and variability in clinical opinion. In contrast, for non‑elite athletes awaiting surgery, extensive rehabilitation with reassessment prior to operative intervention was consistently supported, reflecting recognition of rehabilitation’s potential to influence the need for surgical management.

When determining the most appropriate management strategy, expert surgeons identified concomitant injury, knee stability, and patient choice as key influences on decision making, whereas patient age and level of sport were ranked as less important, challenging traditional assumptions within the literature. However, consensus was not reached regarding secondary complications, graft selection, or the use of lateral extra‑articular procedures, highlighting persistent areas of uncertainty and the need for further high‑quality research.

## Supplementary Information

Below is the link to the electronic supplementary material.

Supplementary file1 (PDF 133 KB)

Supplementary file2 (PDF 115 KB)

Supplementary file3 (PDF 102 KB)

Supplementary file4 (PDF 155 KB)

Supplementary file5 (PDF 107 KB)

Supplementary file6 (XLSX 40 KB)

Supplementary file7 (PDF 104 KB)

## Declarations

### Data Availability

Data supporting the findings of this study are available in Supplement 6 in the electronic supplementary material.

### Patient and Public Involvement

Patients and the public were not involved in the design, conduct, reporting, or dissemination of this study, beyond piloting the questionnaire with an individual who met the study’s inclusion criteria.

### Code Availability

The R code used to for data visualisation is presented in Supplement 5.
