# Supplementary material for: Factors Influencing Consultant Knee Surgeons’ Decision Making in Anterior Cruciate Ligament (ACL) Injury Management in Athletes: An International Delphi Study
Source: Sports Med. 2026 Jun 25;56(10):2611–23. doi: 10.1007/s40279-026-02480-x (PMC13615100; doi:10.1007/s40279-026-02480-x)
Supplement: Supplementary file 1 [file 40279_2026_2480_MOESM1_ESM.pdf]

Supplement 1

Title: Factors influencing consultant knee surgeons' decision making in anterior cruciate ligament (ACL) injury management in athletes: an international Delphi study

Short title: Decision making among expert surgeons in ACL management: A Delphi Study

Authors: Greg Young[1], Nick Dobbin[1,2], Neil Jain[3], Cari Thorpe[1,2]

Affiliation:
[1]Department of Health Professions, Faculty of Health and Education, Manchester Metropolitan University, Manchester, UK, M15 6GX
[2]Institute of Sport, Manchester Metropolitan University, Manchester, UK, M15 6GX
[2]Department of Orthopaedics, The Wilmslow Hospital, 52 Alderley Road, Wilmslow, UK, SK9 1NY

Corresponding Author: Mrs Cari Thorpe, Department of Health Professions, Manchester Metropolitan University, Manchester, UK
Work Tel: 0161 247 2521
Work Email: M.Thorpe@mmu.ac.uk

Alignment of Evidence with Questionnaire Items

Study	Context	Question Alignment
Filbay SR et al. “The only way you’re ever going to play sport again is if you go through surgery”: a mixed methods study exploring anterior cruciate ligament (ACL) injury treatment decisions from the perspective of 734 patients. J Sci Med Sport. 2025;28(10):821-32. Available from: doi: 10.1016/j.jsams.2025.05.010	An Australia-wide study exploring patient beliefs, information received from clinicians, and factors influencing treatment decisions for ACL injuries.	5, 7, 8, 9, 10, 11, 12, 14, 15, 17, 20
Filbay SR, Grindem H. Evidence-based recommendations for the management of anterior cruciate ligament (ACL) rupture. Best Pract Res Clin Rheumatol. 2019;33(1):33-47. Available from: https://doi.org/10.1016/j.berh.2019.01.018	Clinical review providing recommendations for ACL management, discussing rehabilitation as first-line treatment vs surgical reconstruction and associated outcomes.	5, 6, 7, 8, 9, 15, 16, 17, 18, 20
McDermott ID. Anterior cruciate ligament reconstruction: current concepts and latest thinking. Orthop Trauma. 2023;37(3):195-206. Available from: https://doi.org/10.1016/j.mporth.2023.03.008	A review article discussing diagnosis, the decision-making process (conservative vs surgery), graft options, and outcomes of ACL management.	5, 6, 7, 8, 9, 13, 14, 15, 16, 18, 19
Dubé MO, et al. Traumatic knee injury healthcare pathways and outcomes: the Australian knee injury inception cohort study (KIICS) protocol. BMJ Open Sport Exerc Med. 2025;11(4):e002983. Available from: https://doi.org/10.1136/bmjsem-2025-002983	Protocol for a nationwide inception cohort study describing traumatic knee injury healthcare pathways and management strategy predictors in Australia.	3, 4, 5, 9, 10, 11, 12, 13, 14, 18, 20
McIntosh C, et al. The efficacy of anterior cruciate ligament reconstruction and rehabilitation compared to conservative management for reducing the incidence of post-traumatic osteoarthritis following anterior cruciate ligament injury: A systematic review. N Z J Physiother. 2024;52(2):339. Available from: https://doi.org/10.15619/nzjp.v52i2.339	A systematic review comparing the efficacy of ACL reconstruction plus rehabilitation versus rehabilitation alone for reducing the incidence of post-traumatic osteoarthritis.	5, 8, 9, 11, 12, 15, 16, 17
Walker PB, et al. Disparities in ACL injury and management: the impact of sex, race, and social determinants of health. Curr Rev Musculoskelet Med. 2026;19(1):25. Available from: https://doi.org/10.1007/s12178-026-10021-2	Reviews how sex, race, and socioeconomic determinants of health (public vs private/social deprivation) influence injury risk and surgical outcomes.	4, 6, 9, 10, 11, 12
Filbay SR, et al. Healing of acute anterior cruciate ligament rupture on MRI and outcomes following non-surgical management with the cross bracing protocol. Br J Sports Med. 2023;57(23):1490-97. Available from: doi: 10.1136/bjsports-2023-106931	Commentary on surgery versus rehabilitation for ACL injuries, reviewing RCTs like SNNAP, KANON, and COMPARE regarding long-term outcomes and cost-effectiveness.	5, 7, 8, 9, 12, 13, 15, 16, 17
Frobell RB, et al. A randomized trial of treatment for acute anterior cruciate ligament tears. N Engl J Med. 2010;363(4):331-42. Available from: https://doi.org/10.1056/nejmoa0907797	A landmark RCT comparing structured rehabilitation plus early ACL reconstruction against rehabilitation with an optional delayed reconstruction.	5, 7, 8, 15, 16, 17, 18, 20

Frobell RB, et al. Treatment for acute anterior cruciate ligament tear: five year outcome of randomised trial. BMJ. 2013;346;f232. Available from: doi: 10.1136/bmj.f232.		
Reijman M, et al. Early surgical reconstruction versus rehabilitation with elective delayed reconstruction for patients with anterior cruciate ligament rupture: COMPARE randomised controlled trial. BMJ. 2021;372:375. Available from: https://doi.org/10.1136/bmj.n375	A multi-centre RCT from the Netherlands comparing early ACL reconstruction against rehabilitation with optional delayed surgery, focusing on patient perceptions and function.	5, 7, 8, 12, 15, 16, 17
Ardern CL, et al. Fifty-five per cent return to competitive sport following anterior cruciate ligament reconstruction surgery: an updated systematic review and meta-analysis including aspects of physical functional and contextual factors. Br J Sports Med. 2014;48:1543-52.	Systematic review and meta-analysis of 69 articles examining return to sport rates and factors influencing outcomes following ACL reconstruction surgery.	5, 6, 7, 15, 16, 18
Saueressig et al. Primary surgery versus primary rehabilitation for treating anterior cruciate ligament injuries: a living systematic review and meta-analysis. Br J Sports Med. 2022;56(21):1241-51. Available from: doi: 10.1136/bjsports-2021-105359	A living systematic review and meta-analysis comparing primary surgery versus primary rehabilitation, focusing on self-reported function and osteoarthritis.	5, 8, 12, 15, 17
Balboa-Alonso et al. Conservative management of acute anterior cruciate ligament ruptures in recreational athletes: a pilot case series. Cureus. 2026;18(2):e103860. Available from: doi: 10.1177/2325967120964919	A pilot case series of eight recreational athletes investigating the outcomes of conservative management (biologics and rehabilitation) for acute ACL rupture.	5, 7, 8, 9, 15, 16, 17
Filbay SR, et al. Healing of acute anterior cruciate ligament rupture on MRI and outcomes following non-surgical management with the cross bracing protocol. Br J Sports Med. 2023;57(23):1490-97. Available from: doi: 10.1136/bjsports-2023-106931	Investigates MRI evidence of ACL healing and outcomes for non-surgical management using a specific bracing protocol for acute ruptures.	5, 6, 7, 8, 15, 16, 17, 20
Sanders TL, et al. Is anterior cruciate ligament reconstruction effective in preventing secondary meniscal tears and osteoarthritis? Am J Sports Med. 2016;44(7):1699-707. https://doi.org/10.1177/0363546516634325	Retrospective cohort study evaluating if ACL reconstruction prevents secondary meniscal tears and OA, and if timing of surgery affects outcomes.	6, 15, 16, 17, 18, 19
Grindem H, et al. Nonsurgical or surgical treatment of ACL injuries. J Bone Joint Surg Am. 2014;96(15):1233-41. Available from: https://doi.org/10.2106/jbjs.m.01054	Prospective cohort study assessing the relationship between knee reinjury after ACL reconstruction and return to level I sports, timing, and function.	6, 7, 8, 12, 15, 18, 19
Kotsifaki R, King E, Bahr R, Whiteley R. Is 9 months the sweet spot for male athletes to return to sport after anterior cruciate ligament reconstruction? Br J Sports Med. 2025;59(9):667-75. Available from: https://doi.org/10.1136/bjsports-2024-108733	Investigates the safety of return to pivoting sports before or after 9 months post-ACL reconstruction based on objective discharge criteria.	6, 7, 8, 17, 20

Beischer S, Gustavsson L, Senorski EH, Karlsson J, Thomeé C, Samuelsson K, Thomeé R. Young athletes who return to sport before 9 months after anterior cruciate ligament reconstruction have a rate of new injury 7 times that of those who delay return. J Orthop Sports Phys Ther. 2020;50(2):83-90. Available from: https://doi.org/10.2519/jospt.2020.9071	Investigates the association between a second ACL injury and time to return to sport/muscle function in young athletes after primary reconstruction.	3, 6, 7, 8, 18, 19
Waldron K, et al. Anterior cruciate ligament rehabilitation and return to sport: how fast is too fast? Arthrosc Sports Med Rehabil. 2022;4(1):175-9. https://doi.org/10.1016/j.asmr.2021.10.027	Summarizes conservative versus accelerated rehabilitation approaches and return-to-sport criteria following ACL reconstruction.	6, 7, 8, 18, 19, 20
Paterno MV, et al. Incidence of second ACL injuries 2 years after primary ACL reconstruction and return to sport. Am J Sports Med. 2014;42(7):1567-73. Available from: https://doi.org/10.1177/0363546514530088	Prospective cohort study investigating the incidence of second ACL injuries within 24 months after primary reconstruction and return to sport.	6, 8, 18, 19, 20
Nematollahi M, et al. A new approach to differentiate those with anterior cruciate ligament deficient knees into copers and non-copers. Physiother Pract Res. 2016;37(2):73-8. Available from: https://doi.org/10.3233/ppr-160072	Verifies whether KOOS subscales can differentiate ACL-deficient patients into copers and non-copers for treatment planning.	6, 7, 17, 20
Herbst E, et al. Functional assessments for decision-making regarding return to sport following ACL reconstruction. Part II: clinical application of a new test battery. Knee Surg Sports Traumatol Arthrosc. 2015;23(5):1283-91. Available from: doi: 10.1007/s00167-015-3546-3	Clinical application of a novel functional test battery for decision-making regarding return to sports following ACL reconstruction.	6, 7, 18, 19, 20
Wang LJ, et al. Post-traumatic osteoarthritis following ACL injury. Arthritis Res Ther. 2020;22(1):57-65. https://doi.org/10.1186/s13075-020-02156-5	Review outlining the incidence, mechanisms, and role of ACL reconstruction in preventing post-traumatic osteoarthritis following injury.	6, 15, 16, 17, 20
Kessler MA, et al. Function, osteoarthritis and activity after ACL-rupture: 11 years follow-up results of conservative versus reconstructive treatment. Knee Surg Sports Traumatol Arthrosc. 2008;16(5):442-8. Available from: https://doi.org/10.1007/s00167-008-0498-x	Retrospective study comparing function, OA, and activity 11 years after ACL rupture treated conservatively versus reconstructive surgery.	5, 15, 16, 17
Korpershoek JV, et al. Does anterior cruciate ligament reconstruction protect the meniscus and its repair? A systematic review. Orthop J Sports Med. 2020;8(7):232596712093389. Available from: https://doi.org/10.1177/2325967120933895	A systematic review evaluating whether ACL reconstruction protects the meniscus from secondary injury and the success rates of concomitant repairs.	6, 7, 8, 15, 16
DeFazio MW, et al. Return to sport after ACL reconstruction with a BTB versus hamstring tendon autograft: a systematic review and meta-analysis. Orthop J Sports Med.	A systematic review comparing return-to-sport rates and performance levels between BTB and Hamstring Tendon (HT) autografts.	6, 18, 19

2020;8(12):2325967120964919. Available from: 10.1177/2325967120964919		
Webster KE, Hewett TE. What is the evidence for and validity of return-to-sport testing after anterior cruciate ligament reconstruction surgery? A systematic review and meta-analysis. Sports Med. 2019;49:917-929. Available from: doi: 10.1007/s40279-019-01093-x	A systematic review evaluating the effectiveness of return-to-sport test batteries in predicting safe return to play and reducing the risk of second injuries.	6, 8, 20
