# Supplementary material for: Factors Influencing Consultant Knee Surgeons’ Decision Making in Anterior Cruciate Ligament (ACL) Injury Management in Athletes: An International Delphi Study
Source: Sports Med. 2026 Jun 25;56(10):2611–23. doi: 10.1007/s40279-026-02480-x (PMC13615100; doi:10.1007/s40279-026-02480-x)
Supplement: Supplementary file 3 [file 40279_2026_2480_MOESM3_ESM.pdf]

Supplement 3

Title: Factors influencing consultant knee surgeons' decision making in anterior cruciate ligament (ACL) injury management in athletes: an international Delphi study

Short title: Decision making among expert surgeons in ACL management: A Delphi Study

Authors: Greg Young[1], Nick Dobbin[1,2], Neil Jain[3], Cari Thorpe[1,2]

Affiliation:

[1]Department of Health Professions, Faculty of Health and Education, Manchester Metropolitan University, Manchester, UK, M15 6GX

[2]Institute of Sport, Manchester Metropolitan University, Manchester, UK, M15 6GX

[2]Department of Orthopaedics, The Wilmslow Hospital, 52 Alderley Road, Wilmslow, UK, SK9 1NY

Corresponding Author: Mrs Cari Thorpe, Department of Health Professions, Manchester Metropolitan University, Manchester, UK

Work Tel: 0161 247 2521

Work Email: M.Thorpe@mmu.ac.uk

Round I Questions

Thank you for your interest in this research study. The study aims to determine expert opinion regarding the management of ACL injuries. We aim to find a consensus on the factors that influence your choice of surgical vs conservative management for ACL injuries.

The following survey should take no more than 10-15 minutes to complete. Once analysed, statements where consensus has not been reached, an anonymised version of the responses will be sent out to participants for further review.

A detailed participant information sheet outlining the study can be found here. By progressing through the survey, you will be consenting to take part in the study. Please initial next to each statement to provide your consent.

1. Name
2. Current Job Title
3. Years of experience with management of ACL injuries.
4. Within which country do you carry out the majority of your work?

5. To what extent do you agree or disagree that both rehabilitation and reconstructive surgery are viable options for the management of ACL injuries in elite athletes?

6. Please rank in order of importance when determining your management approach of ACL injuries:
   - Age of athlete
   - Time elapsed since injury
   - Concomitant injuries – (ie meniscal tear, LCL/MCL injury etc)
   - Stability of the knee
   - Type of sport they participate in – for example cutting / pivoting sports vs straight line sports
   - The level of sport undertaken
   - Previous injuries to the same knee
   - Patient preference
   - Other

7. To what extent do you agree or disagree both rehabilitation and reconstructive surgery are viable options for ACL injuries returning to cutting, twisting, or pivoting sports (for example football, basketball, tennis, badminton etc)

8. To what extent do you agree with or disagree that a rehabilitation only approach carries a greater risk in return to play than surgical management.

9. To what extent do you agree or disagree that the patient should have an active role in the decision-making process for management of their ACL injury?
   Please comment further here if you wish to justify your response.

10. To what extent do you agree or disagree that the healthcare setting, private vs public, influences your decision?
    Please comment further here if you wish to justify your response.

11. To what extent do you agree or disagree that the financial implication to the patient is a factor in determining which management strategy is pursued?
    Please comment further here if you wish to justify your response.

12. To what extent do you agree or disagree that the financial implications to the public health care system are a factor in determining which management strategy is pursued?
    Please comment further here if you wish to justify your response.

13. To what extent do you agree or disagree that increasing waiting lists for elective surgery on the public healthcare system would influence your decision around a rehabilitation only approach?
    Please comment further here if you wish to justify your response.

14. To what extend to do agree or disagree that a surgical approach would always be your first management choice if there were no financial implications or waiting times?
    Please comment further here if you wish to justify your response.

15. To what extent to do agree or disagree that the development of secondary complications (such as osteoarthritis) is multifactorial, and the evidence is inconclusive regarding the difference between rehabilitation and reconstructive surgery?
    Please comment further here if you wish to justify your response.

16. To what extent do you agree or disagree that the potential long-term implications, i.e. joint OA, further meniscal injury, in the rehabilitation only-approach out ways your decision to adopt a surgical only approach?
    Please comment further here if you wish to justify your response.

17. Would you feel confident in advising a rehab only approach in the management of ACL injuries.
    Please comment further here if you wish to justify your response.

18. Historically, the management approach to ACL injuries has been focused on reconstruction.
    If you were to choose a surgical approach, which graft do you typically choose?

19. What is your reasoning for the choice of graft?

20. As a surgeon, what do you think are the research priorities regarding ACL injuries in future.
