# Supplementary material for: Factors Influencing Consultant Knee Surgeons’ Decision Making in Anterior Cruciate Ligament (ACL) Injury Management in Athletes: An International Delphi Study
Source: Sports Med. 2026 Jun 25;56(10):2611–23. doi: 10.1007/s40279-026-02480-x (PMC13615100; doi:10.1007/s40279-026-02480-x)
Supplement: Supplementary file 4 [file 40279_2026_2480_MOESM4_ESM.pdf]

Supplement 4

Title: Factors influencing consultant knee surgeons' decision making in anterior cruciate ligament (ACL) injury management in athletes: an international Delphi study

Short title: Decision making among expert surgeons in ACL management: A Delphi Study

Authors: Greg Young[1], Nick Dobbin[1,2], Neil Jain[3], Cari Thorpe[1,2]

Affiliation:

[1]Department of Health Professions, Faculty of Health and Education, Manchester Metropolitan University, Manchester, UK, M15 6GX

[2]Institute of Sport, Manchester Metropolitan University, Manchester, UK, M15 6GX

[2]Department of Orthopaedics, The Wilmslow Hospital, 52 Alderley Road, Wilmslow, UK, SK9 1NY

Corresponding Author: Mrs Cari Thorpe, Department of Health Professions, Manchester Metropolitan University, Manchester, UK

Work Tel: 0161 247 2521

Work Email: M.Thorpe@mmu.ac.uk

Round 2 and 3 Questions

1. To what extent do you agree or disagree that rehabilitation-only is a viable option for managing ACL injuries in an elite athlete?

   Strongly Agree, Agree, Neither Agree nor Disagree, Disagree, Strongly Disagree, Not Applicable.

2. To what extent do you agree or disagree that rehabilitation-only is a viable option for ACL injuries returning to cutting, twisting or pivoting sports?

   Strongly Agree, Agree, Neither Agree nor Disagree, Disagree, Strongly Disagree, Not Applicable.

3. To what extent do you agree or disagree that a rehabilitation approach carries a greater risk of re-injury on return to play when compared with surgical management?

   Strongly Agree, Agree, Neither Agree nor Disagree, Disagree, Strongly Disagree, Not Applicable.

4. To what extent do you agree or disagree that surgery will be required eventually in athletes who have returned to competition following rehabilitation-only?

   Strongly Agree, Agree, Neither Agree nor Disagree, Disagree, Strongly Disagree, Not Applicable.

5. To what extent do you agree or disagree that the patient should have an active role in the decision-making process for management of their ACL injury?

   Strongly Agree, Agree, Neither Agree nor Disagree, Disagree, Strongly Disagree, Not Applicable.

6. To what extent do you agree or disagree that your decision for surgery may change if a period of successful rehabilitation is completed whilst the patient is on the waiting list?

   Strongly Agree, Agree, Neither Agree nor Disagree, Disagree, Strongly Disagree, Not Applicable.

7. To what extent do you agree or disagree that a more definitive return to play timeline is important when considering your choice of management and the overall impact on an athlete's career?

   Strongly Agree, Agree, Neither Agree nor Disagree, Disagree, Strongly Disagree, Not Applicable.

8. To what extent do you agree or disagree that the financial influence of the club/organisation/NGB is considered in your choice of management?

   Strongly Agree, Agree, Neither Agree nor Disagree, Disagree, Strongly Disagree, Not Applicable.

9. To what extent do you agree or disagree that a surgical approach would always be your first-choice management if there were no waiting times?

   Strongly Agree, Agree, Neither Agree nor Disagree, Disagree, Strongly Disagree, Not Applicable.

10. To what extent do you agree or disagree that the development of secondary complications (such as osteoarthritis) is impacted by a rehabilitation only approach?

    Strongly Agree, Agree, Neither Agree nor Disagree, Disagree, Strongly Disagree, Not Applicable.

11. To what extent do you agree or disagree that the development of secondary complications (such as osteoarthritis) is impacted by a surgical only approach?

    Strongly Agree, Agree, Neither Agree nor Disagree, Disagree, Strongly Disagree, Not Applicable.

12. Please rank the following factors in importance from 1 to 5. Rank 1 is most important, Rank 5 is least important. Please note these 5 factors were the most commonly selected in the previous round, and there may be other factors you consider that are not included in this list. Concomitant injuries, patient choice, patient perception of stability of their knee, age of athlete, level of sport undertaken.

    Most important (1) → Least Important (5)

13. Given the debate in the literature / medical community surrounding surgical versus rehabilitation approach, to what extent do you agree or disagree that you would feel confident in referring a patient for a rehabilitation only approach?

    Strongly Agree, Agree, Neither Agree nor Disagree, Disagree, Strongly Disagree, Not Applicable.

14. To what extent do you agree or disagree that autologous BTB grafts are preferential in elite athletes?

    Strongly Agree, Agree, Neither Agree nor Disagree, Disagree, Strongly Disagree, Not Applicable.

15. To what extent do you agree or disagree that autologous hamstring grafts are preferential in elite athletes?

    Strongly Agree, Agree, Neither Agree nor Disagree, Disagree, Strongly Disagree, Not Applicable.

16. To what extent do you agree or disagree that allografts are preferential in elite athletes?

    Strongly Agree, Agree, Neither Agree nor Disagree, Disagree, Strongly Disagree, Not Applicable.

17. To what extent do you agree or disagree that synthetic grafts are preferential in elite athletes?

    Strongly Agree, Agree, Neither Agree nor Disagree, Disagree, Strongly Disagree, Not Applicable.

18. To what extent do you agree or disagree that a lateral extra-articular procedure should be included when operating on elite athletes?

    Strongly Agree, Agree, Neither Agree nor Disagree, Disagree, Strongly Disagree, Not Applicable.

19. If you agreed with the previous statement (Question 19), to what extent do you agree or disagree that your lateral extra-articular procedure of choice would be a lateral extra-articular tenodesis?

    Strongly Agree, Agree, Neither Agree nor Disagree, Disagree, Strongly Disagree, Not Applicable.

20. If you agreed with the previous statement (Question 19), to what extent do you agree or disagree that your lateral extra-articular procedure of choice would be an anterolateral ligament reconstruction?

    Strongly Agree, Agree, Neither Agree nor Disagree, Disagree, Strongly Disagree, Not Applicable.
