# Supplementary material for: Factors Influencing Consultant Knee Surgeons’ Decision Making in Anterior Cruciate Ligament (ACL) Injury Management in Athletes: An International Delphi Study
Source: Sports Med. 2026 Jun 25;56(10):2611–23. doi: 10.1007/s40279-026-02480-x (PMC13615100; doi:10.1007/s40279-026-02480-x)
Supplement: Supplementary file 5 [file 40279_2026_2480_MOESM5_ESM.pdf]

Supplement 5

Title: Factors influencing consultant knee surgeons' decision making in anterior cruciate ligament (ACL) injury management in athletes: an international Delphi study

Short title: Decision making among expert surgeons in ACL management: A Delphi Study

Authors: Greg Young[1], Nick Dobbin[1,2], Neil Jain[3], Cari Thorpe[1,2]

Affiliation:

[1]Department of Health Professions, Faculty of Health and Education, Manchester Metropolitan University, Manchester, UK, M15 6GX

[2]Institute of Sport, Manchester Metropolitan University, Manchester, UK, M15 6GX

[2]Department of Orthopaedics, The Wilmslow Hospital, 52 Alderley Road, Wilmslow, UK, SK9 1NY

Corresponding Author: Mrs Cari Thorpe, Department of Health Professions, Manchester Metropolitan University, Manchester, UK

Work Tel: 0161 247 2521

Work Email: M.Thorpe@mmu.ac.uk

R Code for Data Visualisation

Figure 1

```
library(tidyverse)

# Manually entered summary statistics
summary_df <- tribble(
  ~Question, ~Round, ~Median, ~IQR,
  "Q1",  "R2", 4, 2,
  "Q1",  "R3", 4, 3,
  "Q2",  "R2", 4, 2,
  "Q2",  "R3", 5, 1,
  "Q3",  "R2", 2, 1,
  "Q3",  "R3", 2, 1,
  "Q4",  "R2", 3, 1,
  "Q4",  "R3", 3, 1,
  "Q5",  "R2", 1, 0,
  "Q5",  "R3", 1, 0,
  "Q6",  "R2", 2, 1,
  "Q6",  "R3", 2, 0,
  "Q7",  "R2", 2, 0,
  "Q7",  "R3", 2, 0,
  "Q8",  "R2", 4, 2,
  "Q8",  "R3", 4, 1,
  "Q9",  "R2", 4, 1,
  "Q9",  "R3", 4, 0,
  "Q10", "R2", 3, 1,
  "Q10", "R3", 3, 1,
  "Q11", "R2", 3, 1,
  "Q11", "R3", 3, 1,
  "Q13", "R2", 2, 2,
  "Q13", "R3", 3, 2,
  "Q14", "R2", 3, 2,
  "Q14", "R3", 3, 2,
  "Q15", "R2", 3, 1,
  "Q15", "R3", 3, 1,
  "Q16", "R2", 5, 1,
  "Q16", "R3", 5, 1,
  "Q17", "R2", 5, 1,
  "Q17", "R3", 5, 0,
  "Q18", "R2", 3, 3,
  "Q18", "R3", 2, 2,
  "Q19", "R2", 1, 1,
  "Q19", "R3", 1, 0,
  "Q20", "R2", 5, 2,
  "Q20", "R3", 5, 1
) %>%
  mutate(
    Q1 = pmax(1, Median - IQR / 2),
    Q3 = pmin(5, Median + IQR / 2)
  )
```

```
# Add question text labels
question_labels <- c(
  "Q1" = "Q1 - That rehabilitation-only is a viable option for managing ACL injuries in an elite 
athlete?",
  "Q2" = "Q2 - That rehabilitation-only is a viable option for ACL injuries returning to cutting, 
twisting or pivoting sports (football, basketball, tennis, badminton etc)?",
  "Q3" = "Q3 - That a rehabilitation approach carries a greater risk of re-injury on return to play when 
compared with surgical management?",
  "Q4" = "Q4 - That surgery will be required eventually in athletes who have returned to competition 
following rehabilitation-only?",
  "Q5" = "Q5 - That the patient should have an active role in the decision-making process for 
management of their ACL injury?",
  "Q6" = "Q6 - That your decision for surgery may change if a period of successful rehabilitation is 
completed whilst the patient is on the waiting list?",
  "Q7" = "Q7 - That a more definitive return to play timeline is important when considering your 
choice of management and the overall impact on an athlete’s career?",
  "Q8" = "Q8 - That the financial influence of the club/organisation/NGB is considered in your choice 
of management?",
  "Q9" = "Q9 - That a surgical approach would always be your first-choice management if there were 
no waiting times?",
  "Q10" = "Q10 - That the development of secondary complications (such as osteoarthritis) is 
impacted by a rehabilitation only approach?",
  "Q11" = "Q11 - That the development of secondary complications (such as osteoarthritis) is 
impacted by a surgical only approach?",
  "Q13" = "Q13 - That you would feel confident in referring a patient for a rehabilitation only 
approach?",
  "Q14" = "Q14 - That autologous BTB grafts are preferential in elite athletes?",
  "Q15" = "Q15 - That autologous hamstring grafts are preferential in elite athletes?",
  "Q16" = "Q16 - That allografts are preferential in elite athletes?",
  "Q17" = "Q17 - That synthetic grafts are preferential in elite athletes?",
  "Q18" = "Q18 - That a lateral extra-articular procedure should be included when operating on elite 
athletes?",
  "Q19" = "Q19 - That your lateral extra-articular procedure of choice would be a lateral extra-
articular tenodesis?",
  "Q20" = "Q20 - That your lateral extra-articular procedure of choice would be an anterolateral 
ligament reconstruction?"
)

summary_df <- summary_df %>%
  mutate(
    QuestionText = factor(question_labels[Question], levels = rev(question_labels)),
    Round = factor(Round, levels = c("R2", "R3"))
  )

# Plot
ggplot(summary_df) +
  geom_errorbarh(
    aes(y = QuestionText, xmin = Q1, xmax = Q3, colour = Round),
    height = 0.3, size = 1, position = position_dodge(width = 0.6)
  ) +
  geom_point(
    aes(x = Median, y = QuestionText, colour = Round),
    position = position_dodge(width = 0.6),
    size = 6, shape = 18
```

```
  ) +
  scale_x_continuous(
    breaks = 1:5,
    limits = c(0.5, 5.5),
    labels = c("Strongly agree", "Agree", "Neither", "Disagree", "Strongly disagree")
  ) +
  scale_colour_manual(values = c("R2" = "blue", "R3" = "purple"), name = "Round") +
  labs(x = "Response", y = NULL, title = "") +
  theme_minimal(base_family = "Times New Roman") +
  theme(
    legend.position = "top",
    axis.text.y = element_text(size = 13),
    axis.text.x = element_text(size = 13),
    panel.grid.major = element_blank(),
    panel.grid.minor = element_blank(),
    axis.ticks = element_line(color = "black"),
    axis.line = element_line(color = "black")
  )

ggsave("/Users/nickdobbin/Desktop/LikertPlot.tiff", dpi = 300, width = 18, height = 10.27, units =
"in")
```

Figure 2

```
library(ggplot2)
library(tidyverse)
library(scales)
library(RColorBrewer)

# Define full question labels (same as you have)
question_labels <- c(
  "Q1" = "Q1 - That rehabilitation-only is a viable option for managing ACL injuries in an elite
athlete?",
  "Q2" = "Q2 - That rehabilitation-only is a viable option for ACL injuries returning to cutting,
twisting or pivoting sports (football, basketball, tennis, badminton etc)?",
  "Q3" = "Q3 - That a rehabilitation approach carries a greater risk of re-injury on return to play when
compared with surgical management?",
  "Q4" = "Q4 - That surgery will be required eventually in athletes who have returned to competition
following rehabilitation-only?",
  "Q5" = "Q5 - That the patient should have an active role in the decision-making process for
management of their ACL injury?",
  "Q6" = "Q6 - That your decision for surgery may change if a period of successful rehabilitation is
completed whilst the patient is on the waiting list?",
  "Q7" = "Q7 - That a more definitive return to play timeline is important when considering your
choice of management and the overall impact on an athlete’s career?",
  "Q8" = "Q8 - That the financial influence of the club/organisation/NGB is considered in your choice
of management?",
  "Q9" = "Q9 - That a surgical approach would always be your first-choice management if there were
no waiting times?",
  "Q10" = "Q10 - That the development of secondary complications (such as osteoarthritis) is
impacted by a rehabilitation only approach?",
  "Q11" = "Q11 - That the development of secondary complications (such as osteoarthritis) is
impacted by a surgical only approach?",
```

```
  "Q13" = "Q13 - That you would feel confident in referring a patient for a rehabilitation only approach?",
  "Q14" = "Q14 - That autologous BTB grafts are preferential in elite athletes?",
  "Q15" = "Q15 - That autologous hamstring grafts are preferential in elite athletes?",
  "Q16" = "Q16 - That allografts are preferential in elite athletes?",
  "Q17" = "Q17 - That synthetic grafts are preferential in elite athletes?",
  "Q18" = "Q18 - That a lateral extra-articular procedure should be included when operating on elite athletes?",
  "Q19" = "Q19 - That your lateral extra-articular procedure of choice would be a lateral extra-articular tenodesis?",
  "Q20" = "Q20 - That your lateral extra-articular procedure of choice would be an anterolateral ligament reconstruction?"
)

# Input data (same as yours)
df <- tribble(
  ~Question, ~Round, ~Disagree, ~Agree, ~Overall,
  "Q1",  "R2", 68, 23, 35,
  "Q1",  "R3", 68, 32, 35,
  "Q2",  "R2", 68, 18, 35,
  "Q2",  "R3", 77, 23, 52,
  "Q3",  "R2", 5, 86, 48,
  "Q3",  "R3", 0, 100, 61,
  "Q4",  "R2", 23, 41, 35,
  "Q4",  "R3", 18, 36, 43,
  "Q5",  "R2", 0, 100, 83,
  "Q5",  "R3", 0, 100, 91,
  "Q6",  "R2", 5, 73, 48,
  "Q6",  "R3", 9, 86, 74,
  "Q7",  "R2", 0, 86, 65,
  "Q7",  "R3", 0, 95, 83,
  "Q8",  "R2", 73, 14, 43,
  "Q8",  "R3", 91, 9, 61,
  "Q9",  "R2", 50, 23, 39,
  "Q9",  "R3", 82, 14, 74,
  "Q10", "R2", 23, 36, 39,
  "Q10", "R3", 23, 32, 43,
  "Q11", "R2", 32, 23, 43,
  "Q11", "R3", 14, 27, 57,
  "Q13", "R2", 32, 55, 43,
  "Q13", "R3", 27, 50, 43,
  "Q14", "R2", 32, 45, 35,
  "Q14", "R3", 36, 45, 39,
  "Q15", "R2", 36, 14, 48,
  "Q15", "R3", 45, 9, 43,
  "Q16", "R2", 91, 5, 61,
  "Q16", "R3", 95, 5, 70,
  "Q17", "R2", 100, 0, 70,
  "Q17", "R3", 100, 0, 83,
  "Q18", "R2", 32, 50, 26,
  "Q18", "R3", 18, 64, 39,
  "Q19", "R2", 0, 59, 35,
  "Q19", "R3", 0, 73, 57,
  "Q20", "R2", 50, 9, 39,
  "Q20", "R3", 64, 4, 4
```

```
)

# Pivot longer and create factor with full question text ordered by Q1 to Q20
df_long <- df %>%
  pivot_longer(cols = c(Disagree, Agree, Overall),
               names_to = "Type",
               values_to = "Percentage") %>%
  mutate(
    QuestionText = question_labels[Question],
    QuestionText = str_wrap(QuestionText, width = 75)
  ) %>%
  # Set levels in the order of question_labels (Q1 to Q20)
  mutate(
    QuestionText = factor(QuestionText, levels = str_wrap(question_labels[names(question_labels)],
width = 75))
  ) %>%
  mutate(
    Type = factor(Type,
                  levels = c("Disagree", "Agree", "Overall"),
                  labels = c("Strongly Disagree or Disagree",
                             "Strongly Agree or Agree",
                             "Overall Score"))
  )

# Plot using QuestionText as y-axis, reversed for top-down order
ggplot(df_long, aes(x = Round, y = fct_rev(QuestionText), fill = Percentage)) +
  geom_tile(color = "grey90") +
  facet_wrap(~Type, nrow = 1) +
  scale_fill_gradientn(
    colours = c(
      "#FFB3BA", "#FFD1A9", "#FFFFBA",
      "#B6FFB3", "#BAE1FF", "#D5BAFF", "#E8BAFF"
    ),
    limits = c(0, 100),
    name = "Percentage"
  ) +
  theme_classic() +
  theme(
    text = element_text(family = "Times New Roman", size = 11),
    strip.text = element_text(size = 11),
    strip.background = element_blank(),
    axis.text.x = element_text(angle = 0, hjust = 0.5),
    axis.text.y = element_text(size = 11),
    plot.title = element_text(face = "bold", hjust = 0.4),
    legend.position = "right",
    plot.margin = margin(10, 10, 10, 10)
  ) +
  labs(
    title = "",
    y = "Question"
  )

ggsave(filename = "~/Desktop/ACL_Agreement_Plot.tiff",
       dpi = 300,
       width = 12,    # extra wide for big margin
```

```
height = 9,
units = "in",
device = "tiff")
```
