# Supplementary material for: Factors Influencing Consultant Knee Surgeons’ Decision Making in Anterior Cruciate Ligament (ACL) Injury Management in Athletes: An International Delphi Study
Source: Sports Med. 2026 Jun 25;56(10):2611–23. doi: 10.1007/s40279-026-02480-x (PMC13615100; doi:10.1007/s40279-026-02480-x)
Supplement: Supplementary file 6 [file 40279_2026_2480_MOESM7_ESM.pdf]

Supplement 7

Title: Factors influencing consultant knee surgeons' decision making in anterior cruciate ligament (ACL) injury management in athletes: an international Delphi study

Short title: Decision making among expert surgeons in ACL management: A Delphi Study

Authors: Greg Young[1], Nick Dobbin[1,2], Neil Jain[3], Cari Thorpe[1,2]

Affiliation:

[1]Department of Health Professions, Faculty of Health and Education, Manchester Metropolitan University, Manchester, UK, M15 6GX

[2]Institute of Sport, Manchester Metropolitan University, Manchester, UK, M15 6GX

[2]Department of Orthopaedics, The Wilmslow Hospital, 52 Alderley Road, Wilmslow, UK, SK9 1NY

Corresponding Author: Mrs Cari Thorpe, Department of Health Professions, Manchester Metropolitan University, Manchester, UK

Work Tel: 0161 247 2521

Work Email: M.Thorpe@mmu.ac.uk

Plain Language Summary

A non-systematic review was conducted to inform the statements used Round 1 of the Delphi study. A total of 31 participants completed round 1, with 22 completing all three rounds The experts had an average of 18 years' experience managing ACL injuries and perform a minimum of 40 ACL reconstructions per year. Consensus emerged around several key areas, such as rehabilitation potentially reducing the need for surgery while on waiting lists and the higher re-injury risk associated with rehabilitation alone. Rehabilitation-only was not recommended in elite athletes, particularly those participating in cutting, twisting or pivoting sports despite recent evidence supporting a rehabilitation-first approach given. No consensus was reached on secondary complications or graft choices, signalling the need for further research in these areas.
